# Unfolding Success Factors and Barriers in Adapting Slovenia’s Health Promotion Centre Model to Bergamo Province: A PIET-T Feasibility Assessment with Time-Dependent Care Implications

**DOI:** 10.3390/epidemiologia7010021

**Published:** 2026-02-03

**Authors:** Giacomo Crotti, Antonio Antonelli, Federica Bonomi, Giulio Borghi, Giulia Parisi, Isabella Trezzi, Nicola Rizzardi, Radivoje Pribakovic Brinovec, Maja Zupanc, Alberto Zucchi, Nicoletta Castelli

**Affiliations:** 1 Agency for Health Protection of Bergamo (ATS Bergamo), 24122 Bergamo, Italy; giacomo.crotti@ats-bg.it (G.C.); antonio.antonelli@ats-bg.it (A.A.);; 2 Azienda Socio-Sanitaria Territoriale Bergamo Est (ASST Bergamo Est), 24068 Seriate, Italy; 3 National Institute of Public Health (NIJZ), 1000 Ljubljana, Slovenia

**Keywords:** health promotion centres, primary health care, health promotion, noncommunicable diseases, time-dependent care, acute cardiovascular events, equity

## Abstract

Background/Objectives: Health Promotion Centres (HPCs) in Slovenia represent a European best practice for integrated prevention and health promotion. This study explores the feasibility of adapting the Slovenian HPC model to Bergamo Province, Lombardy, considering local population needs and health system characteristics. Methods: We conducted a qualitative feasibility and policy analysis based primarily on documentary review, complemented by a webinar, a study visit to Slovenia, and expert consultations (conducted in two group discussions) with professionals from ATS (Agenzia Tutela della Salute) Bergamo and local ASST (Azienda Socio-Sanitaria Territoriale) providers. Data were analysed using the PIET-T framework (Population–Intervention–Environment–Transfer). Results: Eight key elements define the Slovenian model: (1) governance and stewardship; (2) structural financing; (3) standardized service portfolio; (4) systematic preventive referrals; (5) integration with primary care and screening; (6) multidisciplinary teams with codified training; (7) community outreach and equity orientation; and (8) information systems and reporting. While Bergamo shares similar demographic and epidemiological profiles, differences in behavioral risk factors, project-based financing, fragmented initiatives, and limited digital integration necessitate adaptation. The comparative assessment highlighted key areas requiring contextual adaptation, including financing mechanisms, organisational coordination, workforce capacity, digital interoperability, and approaches to equity. Conclusions: The Slovenian HPC experience demonstrates the potential of integrated, community-based health promotion. Its adaptation to Lombardy appears feasible if core components are preserved and tailored to local governance, population, and health system conditions. These organisational features may be particularly relevant for time-dependent conditions, such as acute cardiovascular and cerebrovascular events, by potentially supporting more timely risk-factor management and coordination across diagnostic and emergency pathways. Rather than a blueprint for reform, this experience offers useful insights to reinforce prevention and health promotion within the ongoing territorial care reform in Lombardy.

## 1. Introduction

Noncommunicable diseases (NCDs) remain the leading cause of death and disability in the WHO European Region. Every year, the Region faces 1.8 million avoidable deaths from NCDs, including cardiovascular diseases, cancers, chronic respiratory diseases and diabetes. Of these, around 60% are due to preventable risk factors such as tobacco and alcohol use, unhealthy diets, obesity, high blood pressure and physical inactivity, while the remaining 40% could be averted with timely diagnosis and access to quality care [1]. Strengthening health promotion and prevention policies is therefore crucial, as they directly address the root causes of these deaths and can save millions of lives as well as reduce health care costs. Moreover, in settings where promotion

n-centred prevention is systematically integrated within primary care, as in Slovenia, these actions have shown co-benefits beyond chronic disease control, supporting timely diagnosis, reducing avoidable hospital admissions (e.g., asthma and chronic obstructive pulmonary disease 75.8 per 100,000 vs. EU 116.1 in 2021), and indirectly strengthening the management of time-dependent conditions such as acute myocardial infarction and stroke. Between 2007 and 2015, premature mortality from cardiovascular diseases declined by about 19%, while 30-day mortality after acute myocardial infarction fell from 12.3% in 2010 to 7.5% in 2020, reflecting a system capable of ensuring earlier detection and response [2,3,4]. Yet, despite repeated policy commitments, many countries still struggle to deliver systematic and sustained health promotion within primary health care (PHC) settings. Initiatives are often fragmented, short-term, and insufficiently integrated with primary care, leaving the Region off track to meet global NCD targets [5].

Slovenia has progressively consolidated a distinctive approach to prevention and health promotion. Since 2002, Health Promotion Centres (HPCs) have been institutionalized within primary health care (PHC) centres, transforming small health education units into a nationwide, structured model based on a bio-psycho-social framework. Primary health care (PHC) centres, which act as the first point of contact and gatekeepers for patients, host multidisciplinary teams including general practitioners (GPs), paediatricians, gynaecologists, dentists, nurses, midwives, community nurses, clinical pharmacists and physiotherapists. Multidisciplinary teams in HPCs include nurses, physiotherapists, psychologists and, in upgraded HPCs, dietitians and kinesiologists. HPCs provide fee-free lifestyle programmes on nutrition, physical activity, smoking cessation, harmful alcohol use, mental health, obesity and type 2 diabetes, with a stronger focus on vulnerable populations. They deliver counselling, skills training, group workshops and follow-up, systematically linked to preventive check-ups in family medicine practices. Governance is ensured by the Slovenian National Institute of Public Health (NIJZ), which sets guidelines, manuals and training (4). Over the past two decades, HPCs have been recognized by the European Office of WHO as a best practice (BP), supported by strong governance, stable financing through mandatory health insurance, and a nationally standardized portfolio of interventions [4,5]. Although centred on prevention, the HPC model also yields system co-benefits: by improving risk-factor control, health literacy and standardized referral from preventive check-ups, it supports earlier recognition and timely care for time-dependent conditions (e.g., acute coronary syndromes and stroke), helping to avert avoidable acute events [4,5,6]. Evaluations also confirm that HPCs have reduced health inequalities, increased coverage of preventive services [7], enabled earlier identification of risk factors, and contributed to a 19% decline in premature cardiovascular mortality between 2007 and 2015 [5].

In Italy, investments in prevention remain structurally insufficient compared to other European countries, with levels well below the European Union (EU) average [3,8]. This chronic underinvestment weakens the health system’s capacity to reduce the burden of chronic diseases and to strengthen health promotion. Recent research supports the embedding of prevention and health promotion as central operational functions of territorial health services within PHC [9]. Comparative evidence from European PHC reforms shows that success in scaling preventive models depends on institutional alignment and sustained financing, rather than isolated pilot projects [10].

Within this national context, Lombardy represents an emblematic case: although prevention and health promotion are explicitly included in regional plans and in the recent reform of territorial care (Ministerial Decree 77/2022–DM77), their implementation is uneven. The post-pandemic analysis of emergency department overcrowding has further underscored the urgent need for integrated, community-based, and digitally supported models, as envisioned in the ongoing territorial care reform [11]. Community Health Homes (*Case della Comunità*, CdC) and Family and Community Nurses (*Infermieri di Comunità*, IFeC) are central pillars of the ongoing reform, designed to strengthen primary health care also through the integration of prevention and health promotion [12]. Although Bergamo Province, within the Lombardy Region, hosts a wide range of community-driven prevention and health promotion initiatives, their impact is limited by weak coordination, low visibility, and poor integration with primary care services [13]. Recent evidence from other Italian regions highlights how the implementation of DM77 has faced significant workforce and digital infrastructure challenges, with GPs showing resistance to collaborative models and slow adoption of telemedicine [14].

The coexistence of institutional ambition and local fragmentation makes Bergamo an ideal setting to explore how innovative models, such as the Slovenian HPCs, could be adapted to reinforce prevention, expand health promotion, and reduce health inequalities.

## 2. Materials and Methods

### 2.1. Study Design

We conducted a feasibility study to examine the adaptability of the Slovenian HPC model to the Italian context, with a particular focus on opportunities for integrating health promotion and prevention into PHC. The Bergamo Province was chosen as a reference context to be compared with the Slovenian model. This study was designed as a qualitative feasibility and policy analysis. Documentary review constituted the primary analytic material, while expert consultations and knowledge exchange activities were used to support interpretation and contextualisation of findings. This study was organized in three steps, including a documentary review, knowledge exchange and study visit activities, and comparative analysis guided by the PIET-T (Population–Intervention–Environment–Transfer) framework chosen for its systematic approach to assessing the transferability of health interventions across contexts [15]. The PIET-T framework was applied deductively to organise and interpret evidence across the four domains and to identify facilitators, barriers, and adaptation requirements.

### 2.2. Documentary Review

A narrative review of the available literature was undertaken to reconstruct the evolution, governance arrangements, service portfolio, operational tools, and functioning mechanisms of the HPCs model. Sources included national guidelines, evaluation reports, peer-reviewed publications, and literature from institutional and international organizations.

In addition to published material, we analyzed a large body of operational documents provided by the BP owner, the Slovenian National Institute of Public Health (NIJZ). These included referral forms, eligibility criteria, clinical algorithms, training plans, manuals, and patient education materials. While not all were publicly accessible, they offered granular insights into workflows, human resources (HR) requirements, competencies, and monitoring, which were essential to reconstruct the operational mechanisms of the model. A complete list is reported in Supplementary Table S1. Documents were purposively selected based on their relevance to the core domains of the HPC model (governance, financing, service delivery, referral pathways, workforce and training, and monitoring), and on their use in routine implementation and evaluation.

Additional qualitative data were collected through different activities that were organized by the study team. Firstly, a knowledge transfer webinar was held with NIJZ representatives to present the rationale, structure, and outcomes of the Slovenian HPC model. The session lasted approximately 120 min, was video-recorded and notes were taken by the study team. Secondly, a two-day study visit was organized to an operational HPC in Slovenia, providing field understanding of service delivery, organisational processes, and interprofessional collaboration. During the site visit, two semi-structured focus groups were conducted with local teams working in HPCs, focusing on the operational aspects, and with NIJZ representatives, exploring the organizational, governance, monitoring and financial aspects. Inputs from these activities were used to clarify operational processes and to validate and contextualise interpretations emerging from the document analysis.

### 2.3. Comparative Analysis

Structured analysis was conducted using the PIET-T framework, which offers a systematic lens for assessing the transferability of healthcare models. In the first stage, data from the documentary review, webinar, and study visit activities were organised, summarised, and interpreted by the research team to identify the key elements of the Slovenian HPC model. These elements were then mapped onto the four domains of the PIET-T framework—population, intervention, environment, and transfer/adaptation. In the second stage, the mapped themes were systematically compared with the corresponding characteristics of the Bergamo context, allowing us to identify points of convergence, discrepancies, and contextual constraints. In the final stage, findings were synthesized into two complementary outputs: a comparative matrix, which highlights facilitators, barriers, and adaptation requirements across the PIET-T domains; and a SWOT analysis, a widely applied method in healthcare evaluation that distils key success factors and contextual risks into a concise format to support decision-making [16]. The PIET-T framework was applied deductively to organise and interpret evidence across domains and to identify facilitators, barriers, and adaptation requirements. The SWOT analysis was structured as a 2 × 2 matrix, distinguishing internal factors (strengths and weaknesses) from external factors (opportunities and threats). Internal factors summarise key organisational and operational aspects relevant to implementing the HPC model in the Bergamo context, while external factors capture broader contextual enablers and risks that may affect adaptation.

## 3. Results

### 3.1. Identification of Key Elements

Through the application of our methods, we identified eight mutually reinforcing elements at the core of the Slovenian HPC model. These elements, together with their structured analysis using the PIET-T framework, are presented in Supplementary Table S2, and are further elaborated with reflections on their relevance and possible adaptation within the Lombardy and Bergamo context.

- Governance and national stewardship: The Slovenian HPC model is grounded in and governed through strong national stewardship: the Ministry of Health and the National Institute of Public Health provide strategic direction, standards, and codification to ensure equity and coherence. At the same time, PHC facilities are municipally owned, which affords local autonomy in implementation and responsiveness but can also lead to fragmentation. In Italy, governance is more decentralized, yet local coordination efforts suggest that progressive alignment is achievable when a both flexible and structured system is implemented.
- Structural financing and universal free access: In Slovenia, health promotion and prevention were structurally financed through the compulsory health insurance scheme, which fully covers preventive check-ups and lifestyle interventions delivered in HPCs. Financial incentives are also provided to general practitioners to encourage the uptake of preventive activities. This stable funding framework ensures that services are free at the point of use and available to the whole population, including vulnerable groups. In Italy, prevention is formally recognized as an Essential Level of Care (LEA), but in practice it is often supported through short-term, project-based resources (e.g., Piano Regionale di Prevenzione—PRP, Piano Nazionale di Ripresa e Resilienza—PNRR). This contrast underscores the need to move toward stable, multi-year financing mechanisms in order to guarantee equity and sustained improvements in population health outcomes.
- Standardised and codified service portfolio: In Slovenia, the HPC portfolio is standardised and highly codified across the services offered, the staffing profiles within HPCs, and the training requirements. This high degree of codification is supported by an extensive corpus of operational documents (Supplementary Table S1), which define workflows, competencies, and monitoring procedures. Such standardisation ensures fairness and consistency nationwide, but could limit responsiveness to emerging needs. In Lombardy, where initiatives are diverse but largely uncoordinated, systematic mapping and certification of activities could help build a coherent portfolio while preserving the flexibility required to address local priorities such as youth mental health, gambling, and other emerging issues.
- Systematic referral through preventive check-ups: In Slovenia, systematic preventive check-ups by GPs or family practice nurse and the standardized referral pathways to HPCs for structured free of charge lifestyle interventions are a central feature of the model and are sustained by strong evidence of effectiveness. Although initial skepticism among GPs due to the additional workload, targeted training and financial incentives facilitated their engagement. Italy lacks an equivalent mechanism, but the development of digital tools (e.g., electronic health records), profs of effectiveness and possibly incentive schemes for GPs could enable a gradual adaptation and transfer of the element.
- Integration with primary care and screening programs: Embedding HPCs in PHC and linking them with screening programs has produced measurable results in Slovenia. In Lombardy, screening programs are strong but only partially connected with primary care; CdC could serve as the institutional hub for integration.
- Multidisciplinary teams supported by codified training: Codified staffing and training in Slovenia ensure consistency, but shortages persist. In Lombardy, DM77 provides a structural basis for multiprofessional teams, though implementation is uneven. Adaptation will require investment in training and innovative strategies such as peer education and community empowerment.
- Community outreach and equity orientation: Slovenia’s explicit equity mandate highlights the importance of outreach to migrants, youth, and vulnerable groups. While Bergamo’s diverse community networks represent a significant asset, ensuring continuity and consistency requires structured coordination and the formal integration of equity as a system-level objective.
- Information systems and reporting: National indicators and digital platforms in Slovenia create transparency, despite interoperability gaps. In Lombardy, fragmented regional systems continue to pose a significant barrier. Strengthening interoperability and implementing shared indicators are essential prerequisites for coherent and effective health system monitoring.

### 3.2. PIET-T Comparative Analysis

The analysis focused on the key dimensions of the PIET-T framework: Population, Intervention, Environment, Transfer, highlighting key insights and their implications for adapting the model to the Bergamo context.

Population: Slovenia and Bergamo share broadly comparable demographic and epidemiological profiles, characterized by ageing populations (≥65 years: 21.1% in Slovenia, 24% in Bergamo) and a high burden of NCDs. In Slovenia, circulatory diseases (33%) and cancers (27.2%) are the main causes of death, while in Bergamo, cancers represent an even larger share (33.4% male, 31.6% female deaths) with cardiovascular disease at around 27%. Evidence from Slovenia shows that the introduction of HPCs contributed to a 19% reduction in premature cardiovascular mortality between 2007 and 2015. Behavioral risks show divergence: adult obesity (19.4%) and heavy drinking (22.7%) are higher in Slovenia, while Bergamo faces physical inactivity (70% of adults), childhood overweight (19%), and adolescent gambling as prominent challenges. Migrant populations are also slightly larger in Bergamo (11% vs. 7.4% in Slovenia). These differences suggest that while the standardized HPC portfolio addresses population-wide risks, adaptations in Bergamo should emphasize culturally sensitive approaches and youth-focused priorities such as mental health and gambling.

Intervention: Slovenia’s model is highly codified, with national guidelines, manuals, and competency frameworks regulating team activities and referral pathways. Interventions are evidence-based, covering cardiovascular prevention, diabetes, mental health, stress management, lifestyle counselling, and group workshops. Systematic referral through preventive check-ups ensures broad coverage: between 2002 and 2020, more than half of the adult population underwent screening for lifestyle-related risk factors, with referrals directed to appropriate HPC activities. Delivery is supported by multidisciplinary teams (nurses, physiotherapists, psychologists, dietitians, kinesiologists), whose training is centrally defined and codified. In Bergamo province, preventive initiatives are numerous but fragmented, often organized by schools, municipalities, or third-sector actors and not systematically integrated with primary care. They are also not always aligned with the directives of the PRP, and a recurrent weakness is the limited evidence base underpinning the effectiveness of many interventions. Moreover, the one-off (“spot”) nature of considerable activity undermines sustainability over time and fails to ensure equitable access across population groups. Therefore, aligning contents with local priorities (e.g., youth gambling, migrant health, mental health) and with the PRP, while building workforce capacity to deliver structured, standardized, and evidence-based interventions, stands as paramount.

Environment: Slovenia benefits from a small, centralised health system, strong political commitment, and structural financing through the Health Insurance Institute. HPCs were introduced in 2002 as part of the cardiovascular disease (CVD)Prevention Programme and later expanded using Norwegian (2013–2016) and EU cohesion funds (2017–2020). This trajectory built on earlier prevention programmes and health education units, within a context of well-established public health traditions. Service organization, however, remains complex due to municipal ownership of Community Health Centres (CHCs), which contributes to IT fragmentation and uneven service availability. Since 2008, NIJZ has provided national leadership, including training, surveillance, and evaluation. In contrast, Italy operates a decentralized, multilayered governance system (State, Region, ATS, ASST, municipalities), which limits coherence. In Lombardy and Bergamo, prevention is recognised as a core mandate (LEA) but often financed through time-limited projects (PNRR, PRP). The development of CdC and the 2025 Local Integrated Health Promotion Plan represent important opportunities for institutional anchoring. However, coordination remains heterogeneous.

Transfer: Insights from the Slovenian HPC experience are most valuable when adapted to the Italian context rather than simply replicated. A key condition for the integration of health promotion into primary care is embedding financing within stable, multi-year frameworks. These frameworks should move beyond project-based PNRR and PRP resources. Equally important is the development of interoperable digital systems linked to the regional electronic health record. Systematic training for primary care and community staff is also essential. The Slovenian model highlights the importance of codified guidance combined with political commitment, stakeholder engagement, and cultural alignment. In Italy, this translates into the need to balance standardization with flexibility so that national priorities can be reconciled with specific local needs. Enabling conditions are beginning to emerge in Lombardy, including the reform introducing CdC, the role of IFeC, and the Local Integrated Health Promotion Plan (PIL 2025). Nevertheless, persistent gaps in financing, governance, and workforce must be addressed to ensure sustainable implementation.

Overall, the Slovenian HPC model should be viewed not as a blueprint to copy, but as a set of structured elements and lessons that can inform Italy’s reform trajectory. Adaptation in Bergamo requires balancing codification and flexibility, combining stable financing, digital integration, workforce strategies, and explicit equity goals.

### 3.3. SWOT Analysis

The comparative PIET-T analysis identified both strengths and constraints for the potential adaptation of the Slovenian Health Promotion Centre model to Bergamo. To provide a concise overview, findings were synthesized into a SWOT matrix (Table 1). SWOT elements were derived from the PIET-T–guided comparative analysis and expert consultations, and synthesised by the research team to summarise internal conditions and external enablers/risks affecting model adaptation. This analysis highlights the main assets of the Slovenian model: strong governance, structural financing, synthesized service portfolio and the vulnerabilities of the Italian context (fragmented governance, project-based funding and workforce shortages). At the same time, it underscores opportunities linked to the ongoing territorial reform in Lombardy (CdC, IFeC) and the risks posed by structural barriers (limited interoperability, shortage of trained staff).

## 4. Discussion

The findings presented in this section are grounded in documentary analysis and expert consultations. Broader implications and policy-oriented considerations are explicitly presented as interpretative reflections informed by these empirical materials. Overall, this feasibility and policy analysis suggests that the Slovenian HPC model offers a structured approach to health promotion and prevention. Its potential adaptation to Italy would require balancing core standardised components with context-specific flexibility. The comparative PIET-T analysis confirms that while demographic and epidemiological profiles align well between Slovenia and Bergamo, differences in behavioral risk factors, governance structures, and financing mechanisms shape the conditions for successful adaptation. The value of applying the PIET-T framework lies in its ability to go beyond surface comparability. As emphasized by Schloemer and colleagues [15,17], transferability cannot be assumed; it requires systematic consideration of the population, the intervention, and the environment, along with the readiness of the receiving context. Our findings confirm this principle: population-level comparability is high, but environmental fit is only moderate due to decentralized governance and project-based financing in Italy. This reinforces that adaptation of successful key elements, rather than replication, is the feasible pathway.

The TO-REACH project (Transfer of Organizational innovations for Resilient, Effective, equitable, Accessible, sustainable and Comprehensive Health services and systems)recommendation on cross-national learning further strengthens this perspective. It argues that innovations cannot simply be “copied” across borders but must be translated and tailored to local contexts [18]. Our study illustrates this principle. The Slovenian BP provides a robust backbone of governance, financing, and referral mechanisms, alongside an unusually extensive corpus of codified tools, including operational manuals, training modules, and clinical algorithms (Supplementary Table S1), representing a rare level of specification in health promotion that clarifies “what to do, by whom, and how.” Such codification supports fidelity and scalability; however, as the Organisation for Economic Co-operation and Development (OECD) Guidebook on Best Practices in Public Health reminds us, documents alone are insufficient to secure successful adoption [19]. Political commitment, stakeholder engagement, and cultural alignment in the receiving system are equally decisive. For Lombardy, codified materials are a valuable entry point, but their usefulness ultimately depends on coupling them with local capacity-building and contextual adaptation within the ongoing territorial reform.

Equity is another domain where broader literature reinforces our findings [5]. Slovenia’s HPCs have equity explicitly embedded in their mandate through outreach, intercultural mediation, and targeted group programs. An equity-oriented promotion system also contributes to timeliness of care: by engaging vulnerable and hard-to-reach groups earlier, HPCs not only reduce health gaps but also shorten delays in seeking help for time-dependent conditions, improving early access to diagnostic and emergency pathways [20]. The PIET-T framework stresses that contextual variation in population vulnerabilities must inform adaptation decisions.

Bergamo’s higher share of foreign residents and specific youth vulnerabilities illustrate why equity must not be left implicit. It should be declared a system-level objective, monitored through shared indicators. An integrated approach to reducing the burden of chronic diseases plays a key role across European health systems. A lack of coordination in chronic disease management often leads to acute, time-dependent events such as myocardial infarction and stroke, increasing pressure on emergency services. Multidisciplinary, promotion-oriented models like the HPCs address these gaps by combining early risk identification, lifestyle counselling, and patient empowerment, thereby reducing escalation into acute crises. Recent Italian evidence shows that improving integration across care levels and enhancing population health literacy can shorten pre-hospital delays and improve outcomes for stroke and STEMI patients [10]. Strengthening people’s capacity to recognise early warning signs and access care promptly—through structured health promotion and education—complements the emergency system and contributes to better outcomes in time-critical conditions.

Finally, the information systems dimension underscores the broader challenge of systemic adaptation. Slovenia’s experience shows both the benefits and the limits of digital infrastructure: nationally defined indicators provide a common monitoring framework, yet municipal IT fragmentation constrains integration. Italy faces similar, but more pronounced, heterogeneity across regions. Strengthening primary health care and health promotion will require interoperable systems, to support the recording of a core set of shared indicators, transparent reporting, all embedded in a broader capacity-building effort.

Evidence from our analysis, undertaken to inform the ongoing reform of Italy’s PHC system locally, suggests that adopting selected high-impact components from the mature Slovenian best practice (systematic preventive health checks, codified referral pathways, stable multiyear financing, multidisciplinary teams, and standardized training), while tailoring delivery to local priorities and governance arrangements, is feasible and could be part of a broader strategy in the effort to strengthen primary care and health promotion. The Slovenian experience demonstrates that prevention can be institutionalized; the Italian challenge lies in achieving comparable sustainability and equity within a more fragmented system.

This study is exploratory and qualitative. It relied on desk review, BP owner engagement, and a site visit, without quantitative data collection in Italy. A further limitation is that a substantial share of the evidence base comprised internal and working documents provided by NIJZ. These materials offered essential operational details but they may introduce provider bias and are not fully accessible to the broader scientific community. To mitigate this, we triangulated them with WHO, OECD, and Health Systems in Transition (HiT) reports and validated findings with Italian stakeholders. By listing all sources in Supplementary Table S1, we enhance transparency and reproducibility.

## 5. Conclusions

The Slovenian Health Promotion Centres represent a mature best practice with measurable health impact. Bergamo emerges as a promising context for adaptation owing to its active community sector and ongoing territorial reform. The success of adaptation depends on preserving core features—structural financing, referral, multidisciplinary teams, integration with screening, monitoring—while adapting thematic content, workforce, financing mechanisms, and partnership models to local realities.

The extensive documentary backbone (Supplementary Table S1) strengthens feasibility, as it provides detailed tools for training, delivery, and monitoring. At the same time, it requires careful governance to ensure transparency and accessibility. Consistent with PIET-T and TO-REACH frameworks, the process should be understood as translation rather than transfer. By combining codified tools with tacit knowledge and capacity building, Italy has the opportunity to move from fragmented initiatives to an institutionalised, equitable, and sustainable system of health promotion.

## Figures and Tables

**Table 1:** SWOT matrix.

Strengths (Internal Elements Enabling Integration)	Weaknesses (Internal Constraints)
Demographic and epidemiological fit: ageing population and high burden of noncommunicable diseases support the relevance of an HPC-like approach.	Fragmentation of governance across multiple levels (regional and local), limiting coherence and accountability.
Reform context (DM77) already foresees Community Health Homes (CdC) and multiprofessional teams, providing an organisational platform for integration.	Workforce shortages and lack of codified and standardised training pathways for prevention and health promotion.
Strong local prevention culture and planning capacity (e.g., Bergamo PIL 2025).	Prevention activities often financed through project-based resources, reducing continuity and long-term sustainability.
Rich network of community initiatives and civil society engagement supporting outreach and equity-oriented actions.	Fragmented IT systems and limited integration of prevention activities within digital infrastructures and reporting systems.
**Opportunities** **(external enablers)**	**Threats** **(external risks)**
Systematic mapping and light accreditation of existing initiatives could improve coherence, quality, and visibility of prevention activities.	Risk of replicating rigidity of highly codified models, with limited flexibility to respond to emerging local needs.
Expansion of the Electronic Health Record (FSE) and digital infrastructure can support systematic referral pathways and monitoring.	Decentralised governance may slow institutional embedding and standardisation across settings.
EU/PNRR funding can pilot HPC-like mechanisms if linked to structural, multi-year financing models.	Political and financial discontinuity may hinder long-term sustainability beyond project cycles.
Focus on youth mental health, gambling, and lifestyle-related risks aligns local priorities with the core areas addressed by the HPC model.	Resistance from professional groups (e.g., general practitioners) if incentives and workload implications are not adequately addressed.

## Data Availability

The data presented in this study are available on request from the corresponding author. All documents and materials used for the analysis are listed in Supplementary Table S1. Internal documents, notes collected during the knowledge exchange meetings, site visits, and focus groups are not publicly available due to privacy and confidentiality considerations, but are available from the corresponding author upon reasonable request.

## Supplementary Materials

The following supporting information can be downloaded at: https://www.mdpi.com/article/10.3390/epidemiologia7010021/s1, Table S1: Documents reviewed; Table S2: PIET-T framework.

## Abbreviations

The following abbreviations are used in this manuscript:

ASST	Azienda Socio Sanitaria Territoriale—Territorial Social and Health Authority
ATS	Agenzia di Tutela della Salute—Health Protection Agency
BP	Best Practice
CdC	Case della Comunità—Community Health Homes
CHC	Community Health Centre
COPD	Chronic Obstructive Pulmonary Disease
CVD	Cardiovascular Disease
DM77	Decreto Ministeriale 77/2022—Ministerial Decree 77/2022
EU	European Union
GP	General Practitioner
HiT	Health Systems in Transition
HPC	Health Promotion Centre
HR	Human Resources
IFeC	Infermieri di Comunità—Family and Community Nurses
IT	Information Technology
LEA	Livelli Essenziali di Assistenza—Essential Levels of Care
NIJZ	Nacionalni Inštitut za Javno Zdravje—Slovenian National Institute of Public Health
NCD	Noncommunicable Disease
OECD	Organisation for Economic Co-operation and Development
PHC	Primary Health Care
PIET-T	Population–Intervention–Environment–Transfer (framework)
PIL	Piano Integrato Locale—Local Integrated Plan
PNRR	Piano Nazionale di Ripresa e Resilienza—National Recovery and Resilience Plan
PRP	Piano Regionale di Prevenzione—Regional Prevention Plan
STEMI	ST-Elevation Myocardial Infarction
SWOT	Strengths, Weaknesses, Opportunities, Threats (analysis method)
TO-REACH	Transfer of Organizational innovations for Resilience, Effectiveness, And efficiency across health care systems
WHO	World Health Organization
